# Transgenic neuronal overexpression reveals that stringently regulated p23 expression is critical for coordinated movement in mice

**DOI:** 10.1186/1750-1326-6-87

**Published:** 2011-12-28

**Authors:** Ping Gong, Jelita Roseman, Celia G Fernandez, Kulandaivelu S Vetrivel, Vytautas P Bindokas, Lois A Zitzow, Satyabrata Kar, Angèle T Parent, Gopal Thinakaran

**Affiliations:** 1 Departments of Neurobiology, Neurology, and Pathology, The University of Chicago, Chicago, IL, 60637, USA; 2 Committee on Neurobiology, The University of Chicago, Chicago, IL, 60637, USA; 3 Department of Neurobiology, Pharmacology, and Physiology, The University of Chicago, Chicago, IL, 60637, USA; 4 Department of Surgery, The University of Chicago, Chicago, IL, 60637, USA; 5 Departments of Medicine and Psychiatry, Centre for Prions and Protein Folding Diseases, University of Alberta, Edmonton, AB, T6G 2R7, Canada

**Keywords:** p24, gamma-secretase, Alzheimer's disease, ataxia, hypomyelination, unfolded protein response

## Abstract

**Background:**

p23 belongs to the highly conserved p24 family of type I transmembrane proteins, which participate in the bidirectional protein transport between the endoplasmic reticulum and Golgi apparatus. Mammalian p23 has been shown to interact with γ-secretase complex, and modulate secretory trafficking as well as intramembranous processing of amyloid precursor protein in cultured cells. Negative modulation of β-amyloid production by p23 in cultured cell lines suggested that elevation of p23 expression in neurons might mitigate cerebral amyloid burden.

**Results:**

We generated several lines of transgenic mice expressing human p23 in neurons under the control of *Thy-1.2 *promoter. We found that even a 50% increase in p23 levels in the central nervous system of mice causes post-natal growth retardation, severe neurological problems characterized by tremors, seizure, ataxia, and uncoordinated movements, and premature death. The severity of the phenotype closely correlated with the level of p23 overexpression in multiple transgenic lines. While the number and general morphology of neurons in Hup23 mice appeared to be normal throughout the brain, abnormal non-Golgi p23 localization was observed in a subset of neurons with high transgene expression in brainstem. Moreover, detailed immunofluorescence analysis revealed marked proliferation of astrocytes, activation of microglia, and thinning of myelinated bundles in brainstem of Hup23 mice.

**Conclusions:**

These results demonstrate that proper level of p23 expression is critical for neuronal function, and perturbing p23 function by overexpression initiates a cascade of cellular reactions in brainstem that leads to severe motor deficits and other neurological problems, which culminate in premature death. The neurological phenotype observed in Hup23 mice highlights significant adverse effects associated with manipulating neuronal expression of p23, a previously described negative modulator of γ-secretase activity and β-amyloid production. Moreover, our report has broader relevance to molecular mechanisms in several neurodegenerative diseases as it highlights the inherent vulnerability of the early secretory pathway mechanisms that ensure proteostasis in neurons.

## Background

p23 (also termed TMP21, p24c, or p24δ_1_) belongs to the p24 family of type-I transmembrane proteins, which predominantly localize to cis-Golgi and coated protein complex I (COPI)-coated vesicles. The mammalian p24 family contains 10 proteins that can be grouped into four subfamilies, termed p24α, β, γ, and δ [1,2], whose main function is to regulate anterograde and retrograde transport in the early secretory pathway between the endoplasmic reticulum (ER) and Golgi apparatus [3-7]. Eight of the p24 family proteins, including p23, are ubiquitously expressed in mouse tissues. p23/p24δ_1 _is the only member of the p24δ subfamily in vertebrates, with the exception of an amphibian-specific p24δ_2 _[2]. Embryos with targeted deletion of *Tmed10 *(the gene that encodes p23) die at E4.5 prior to implantation, demonstrating that p23 function is essential for mouse embryonic development [8].

Like other p24 proteins, p23 has four recognizable domains, a Golgi dynamics domain at the N-terminus followed by a coiled-coil domain, a transmembrane domain, and a short cytoplasmic tail. The role of p23 in the budding process of COPI vesicle from the cis Golgi membrane is well characterized [9]. In addition, p24 family proteins are involved in the export of glycosylphosphatidylinositol anchored proteins, and likely other select cargo, into COPII vesicles [7]. Finally, p24 proteins are important for maintenance of the ribbon-like Golgi morphology. When overexpressed in mammalian cells, exogenous as well as endogenous p23 mislocalized to the ER, causing expansion and clustering of smooth ER membranes and fragmentation of Golgi apparatus [5,6,10]. Partial loss of p23 expression in kidney cells of p23^+/- ^mice also caused Golgi apparatus to dilate [8]. Thus, both diminution and overexpression of p23 induce changes in Golgi morphology.

A potential new function for p23 in Alzheimer's disease (AD) pathogenesis emerged from a study that identified p23 as a binding partner of γ-secretase [11]. γ-secretase is a multi-transmembrane enzyme complex made of four essential subunits, presenilin, nicastrin, PEN2, and APH1 [12]. Sequential cleavage of amyloid precursor protein (APP) by β-site APP cleaving enzyme BACE1 and then by γ-secretase generates β-amyloid (Aβ) peptides, which are deposited in senile plaques found in brains of individuals with AD [13]. siRNA knockdown of p23 expression in cultured neuronal and non-neuronal cells enhances secretory trafficking of APP as well increased secretion of soluble APP derivatives and Aβ, suggesting that p23 is a negative modulator of Aβ production [11].

Previously we reported that p23 is widely expressed in major brain areas, and co-localizes in neurons with γ-secretase core subunits presenilin 1 and nicastrin [14]. Interestingly, the steady-state p23 levels decline during postnatal development in rat and mouse brain, and are also reduced in the brains of individuals with AD. Based on these findings, we suggested that age-related decline in p23 expression may be an intrinsic factor that significantly impacts on APP processing and Aβ burden in the aging nervous system [14]. To test whether cerebral Aβ levels can be manipulated by neuronal overexpression of p23, we generated multiple lines of transgenic mice that express human p23 under the control of a neuron-specific promoter. Here, we report that neuronal overexpression of p23 causes severe motor dysfunction, growth retardation, infertility, and early death. Although overt neuronal loss was not detected, marked proliferation of astrocytes, activation of microglia, and thinning of myelinated bundless in brainstem of Hup23 mice indicate that neurons overexpressing p23 were indeed subject to crisis or insult. Our data reveal that regulated p23 expression in neurons is critically important for neuronal function, and our study highlights the risks of neuronal overexpression of p23.

## Results

### Generation of Hup23 transgenic mice and characterization of p23 expression

**Figure 1:**
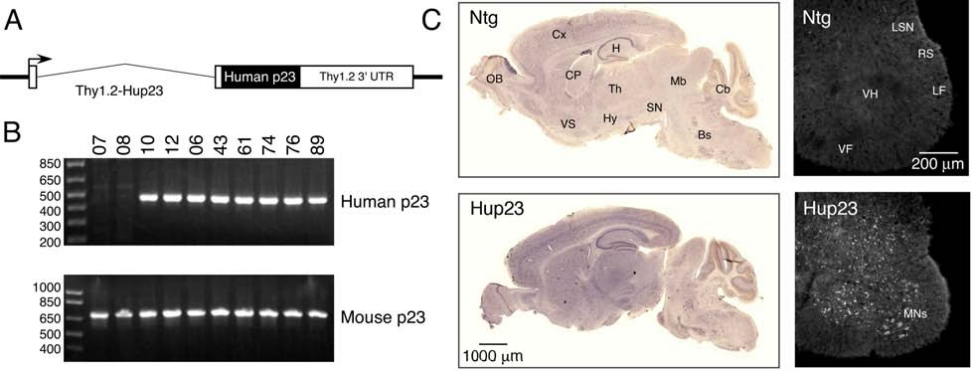
**Generation of Hup23 mice**. ***A***, Schematic representation of the transgenic construct. Human p23 was expressed under the transcriptional control of mouse *Thy-1.2 *promoter using *Thy-1.2 *genomic expression cassette. ***B***, Genotyping of Hup23 mice. Genotyping was performed by PCR of tail DNA using primers specific to human p23 cDNA (upper panel) to amplify a 466-bp PCR product. Control reactions were performed using primers specific for mouse *Tmed10 *locus (lower panel) to amplify 712-bp PCR product. ***C***, The distribution of endogenous and transgene-derived p23. Sagittal brain sections and cross sections of spinal cord from Ntg and Hup23 mice were stained by immunoperoxidase or immunofluorescence labeling, respectively, using polyclonal p23 antibody. In agreement with the previously described pattern of transgene expression from the *Thy-1.2 *genomic expression cassette [15-17], p23 is broadly overexpressed in brain and spinal cord. Brainstem (Bs), caudate putamen (CP), cerebellum (Cb), cortex (Cx), hippocampus (H), hypothalamus (Hy), midbrain (Mb), substantia nigra (SN), thalamus (Th), olfactory bulb (OB), and ventral striatum (VS) are indicated in sagittal brain section. Spinal motoneurons (MNs), lateral spinal nucleus (LSN), rubrospinal tract (RS), lateral funiculus (LF), ventral funiculus (VF), and ventral horn of gray matter (VH) are indicated in spinal cord sections.

**Figure 2:**
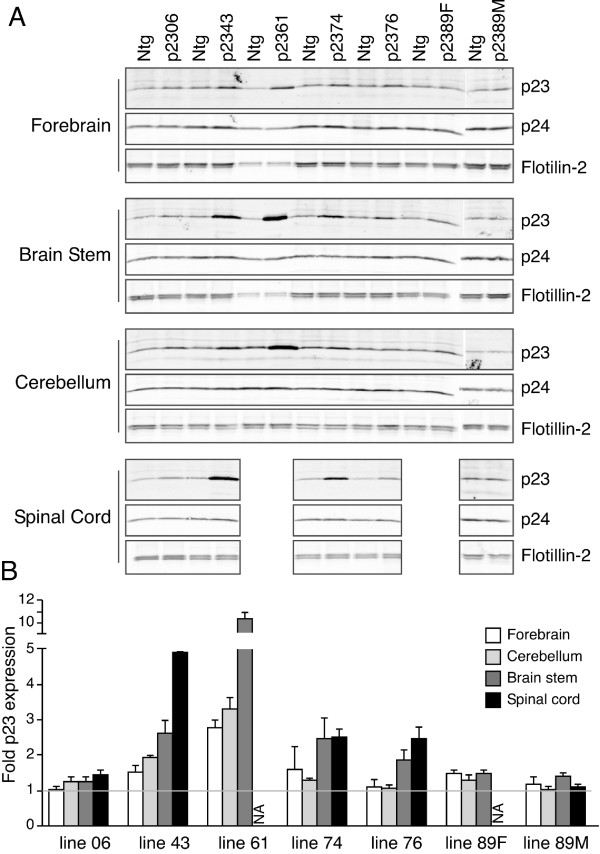
**Overexpression of p23 in forebrain, cerebellum, brainstem, and spinal cord of Hup23 mice**. ***A***, Representative Western blots analysis of steady-state p23 levels in Hup23 mice and littermates. Ninety μg of total protein lysates (or 15 μg in the case of line 61) were fractionated on 4-20% SDS-PAGE gels and immunoblotted using polyclonal p23, p24 and flotillin-2 antibodies. ***B***, Quantification of p23 levels. The signal intensity of p23 from multiple animals from each line (normalized to flotillin-2 signal as loading control) was quantified using Odyssey Imager (LI-COR) and plotted as fold difference in Hup23 mice relative to their Ntg littermates.

In order to overexpress p23 in neurons, we generated transgenic mice expressing human p23 (Hup23 mice) under the transcriptional control of *Thy-1.2 *promoter, which drives neuron-specific expression of transgenes [15-17]. We screened 93 potential founders by PCR, and identified 22 that harbored the human p23 transgene (Figure 1A). By crossing each founder with C57BL/6J mice, we were able to obtain F1 mice harboring the transgene at the expected Mendelian ratio. We then analyzed brain lysates of F1 offspring from all 22 transgene positive potential founders for p23 protein expression. Quantitative Western blots analyses of F1 progeny of all 22 founders revealed neuronal p23 overexpression in brains of only six lines (lines 06, 43, 61, 74, 76, and 89). In these lines p23 was overexpressed to different levels in the forebrain, cerebellum, brainstem, and spinal cord (Figure 2). Although the level of overexpression varied between the six Hup23 lines, it was fairly consistent in all transgenic descendants of each founder.

**Figure 3:**
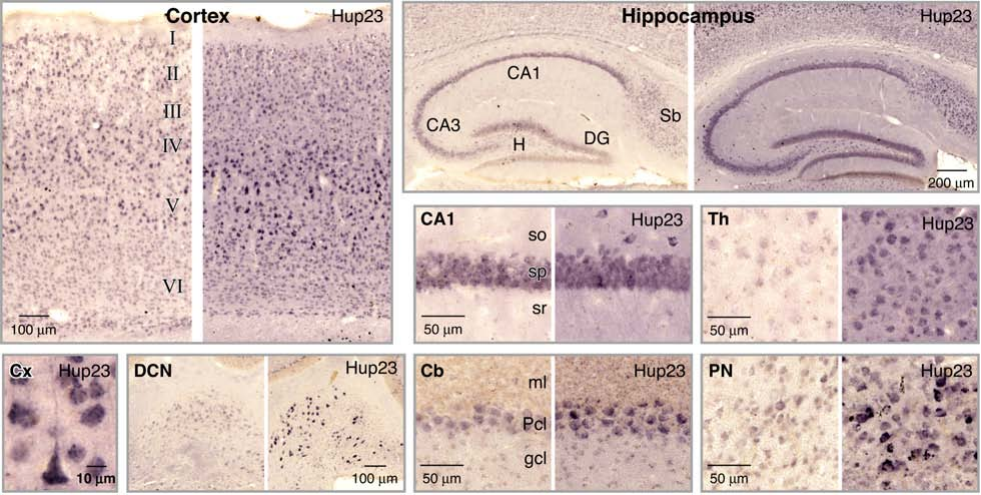
**Immunohistochemical analysis of p23 expression**. Sagittal brain sections of Hup23 Tg mice and Ntg littermates were stained with p23 antibody [14]. Shown are pairs of images from transgenic mouse brain (*Hup23*) and Ntg littermate corresponding to cortex, hippocampus, CA1 region of the hippocampus (CA1), thalamus (Th), deep cerebellar nuclei (DCN), cerebellum (Cb), and pontine nuclei (PN). p23 localizes to the cell body and vertically oriented apical dendrites in Hup23 cortex (Cx). The following areas are indicated: *DG*, dentate gyrus; *gcl*, granule cell layer; *H*, hilus; *ml*, molecular layer; *Pcl*, Purkinje cell layer; *so*, stratum oriens; *sp*, stratum pyramidale; *sr*, stratum radiatum; *Sb*, subiculum.

In previous studies *Thy-1.2 *promoter-driven gene expression was observed in subsets of cortical, hippocampal, cerebellar neurons as well as the majority of motoneurons and sensory neurons [17]. In agreement, immunohistochemical analysis using a highly specific polyclonal p23 antibody [14] showed overall intense p23 staining in brain and spinal cord of Hup23 mice relative to non-transgenic (Ntg) littermates (Figure 1C). As we reported earlier, endogenous p23 is expressed mainly in neurons in rodent brain [14]. Detailed analysis of p23 staining in sagittal sections from Hup23 mice and Ntg littermates revealed p23 overexpression in neurons throughout somatomotor, somatosensory, parietal, and visual cortex, especially in layers IV and V of the cortex (Figure 3). Intense p23 staining was also observed in CA1-CA3 pyramidal neurons of the hippocampus, and in neurons located in the dentate gyrus, hilus, as well as subiculum. Overall staining intensity was also higher in the thalamus, hypothalamus, inferior colliculus, and within the substantia nigra. In the cerebellum, p23 overexpression was evident in cerebellar Purkinje cells, molecular and granule cell layers, and in the deep cerebellar nuclei. Consistent with immunoblot results, higher expression of p23 was apparent throughout the brainstem, particularly in neurons within the pontine nuclei and superior olivary complex (Figures 1 and 3). In the spinal cord, transgene-derived p23 was highly expressed in neurons of all laminae with the exclusion of the superficial laminae 1 and 2. Consistent with previous observations of *Thy-1.2 *promoter-driven transgene expression [17], transgenic p23 expression could be clearly seen in motoneurons in the ventral horn of the spinal cord (Figure 1C).

### Resting tremor, seizure-like activity, motor disorder, and early death in Hup23 mice

Initially, we noticed that transgenic offspring in Hup23 line 06 displayed abnormal movement and had shorter life span. As we generated F1 offspring from additional founders, we observed that Hup23 mice in five additional lines (lines 43, 61, 74, 76, and 89, all of which had p23 overexpression) were born normal but developed a complex motor disorder phenotype with insidious onset few weeks after birth (see Table 1). Animals exhibited hunched posture, head bobbing, uncoordinated and jerky movements, staggered gait, ataxia, exaggerated movement of the tail, periodic knuckling over of the front paws, as well as transient and intermittent resting tremor to occasional seizures. A few of the animals also exhibited prolonged extension of the tarsus and flexing of the front digits (clenching). Additional movie files illustrate these abnormalities [see Additional files 1 and 2]. Every transgenic offspring in each of the six lines where we observed p23 overexpression and the Hup23 founder #43 developed tremor and movement problems. Notably, offspring of remaining 16 potential founders that failed to show p23 overexpression by quantitative immunoblotting or immunostaining were free of any symptoms.

**Table 1:** A comparison of p23 overexpression and the onset of phenotype in Hup23 mice

Line		Fold overexpression	Symptoms observed by DigiGait analysis	Symptoms visually observed	Age at death
06		1 - 1.5	~P30	~P37	~P60

43		1.4 - 4.9	P20	P22	P33 - 34

61		2.8 - 10.4	N.D.	P14	P17 - 21

74		1.3 - 2.5	P28	P32	P43 - 50

76		1.1 - 2.5	P28	P32	P41 - 48

	F	1.3 - 1.5	P33	P30	P42 - 44
89					
	M	1 - 1.4	P40	P36	Survived

N.D. not determined. F, female; M, male.

The onset of the symptoms varied in each transgenic line and ranged between P14 and P40 (Table 1). With the exception of male mice in line 89, all Hup23 Tg mice died shortly after the onset of the phenotype. The time interval between the first visible sign of shaking/shudder phenotype to death varied in each line. In line 61 where the phenotype was most severe, symptoms appeared at P14 and most animals died by P21. In line 06 where the symptom was milder, a subtle phenotype was observed around P37, which deteriorated to a visible phenotype by P45, and death occurred around P60. Because premature death occurred before the mice reached sexual maturity, we were unable to generate Hup23 F2 offspring. The males of Hup23 mice in line 89 were the only ones survived beyond adulthood. Nevertheless, they also failed to breed beyond F1. Therefore, all experiments were performed with F1 animals.

The severity of symptoms and the level of p23 expression appeared to show a high degree of correlation (Table 1; [see Additional file 1]). Hup23 mice in the two lines with the highest p23 overexpression levels, line 43 and 61, developed more severe symptoms, showed earlier onset, and had shorter life span. The levels of p23 overexpression in line 06 and 89 were not high (~1.2 fold in the forebrain and cerebellum and 1.5 fold in the brain stem and spinal cord), and yet were sufficient to cause the impaired movement phenotype.

### Postnatal growth retardation in Hup23 mice

**Figure 4:**
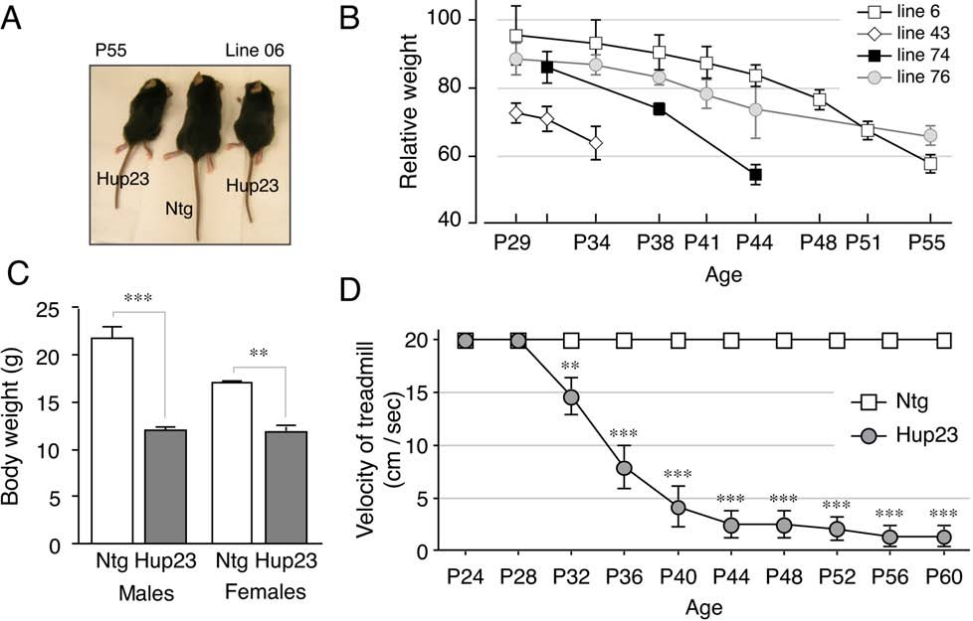
**Movement problem of Hup23 mice**. ***A***, Three male F1 littermates of line 06 at the age of P55 are shown for comparison of body size. ***B***, Progressive decline in body weights in Hup23 animals. The weight of each Hup23 animal was compared to the average weight of its own Ntg littermate(s) of the same sex, and the mean ± SEM of 4-7 animals from each line are plotted. ***C***, The average body weight of Hup23 mice and their littermates in each line on the last day of measurement (see B) is plotted as mean ± SEM. A one-way ANOVA with Tukey post-hoc comparison was performed to assess statistical significance. **, *p *< 0.01; ***, *p *< 0.001. ***D***, DigiGait analysis. The performance of Ntg mice on the DigiGait is shown as a black line with open rectangles. Hup23 mice and littermates were subject to DigiGait trial every other day at treadmill speeds of 20 cm/sec or lower as described under Methods. The animals were tested at lower speeds if they were too impaired to keep up with the treadmill kept at 20 cm/sec. If a mouse failed to gain speed and run when the treadmill was kept at 5 cm/sec, 0 cm/sec is rendered to that trial and the animal was not tested any further. Each black circle represents the average speed at which Hup23 mice can run at a given age. The mean ± SEM of data from 14 Hup23 Tg mice from 4 lines and Ntg littermates is plotted. A one-sample *t*-test was performed to compare the speed of Hup23 mice at each age with to the speed of Ntg animal (20 cm/sec). **, *p *< 0.01; ***, *p *< 0.0001 for all other ages tested.

Newborn Hup23 mice were similar in size and body weight as compared with Ntg littermates of the same sex and weanlings exhibited normal grooming behavior. However, Hup23 mice failed to gain weight at similar rates to their Ntg littermates as they aged. We compared the weight of 14 Hup23 mice from 5 different lines to their Ntg littermates and found that in each line juvenile Hup23 mice were smaller in body size and had lower body weight relative to their Ntg littermates (Figure 4A and 4B). For instance, Hup23 mice from line 74 showed significant weight difference starting at P32 (*p *< 0.05 on P32; *p *< 0.001 on subsequent days). A one-way ANOVA test of animals from four lines revealed significant differences in body weight between Hup23 mice and corresponding Ntg littermates [F (3, 8) = 39.15, *p *< 0.0001]. Tukey post-hoc comparisons showed significantly lower weights in symptomatic transgenic males (*p *< 0.001) as well as females (*p *< 0.01) as compared with non-transgenic littermates of the same sex (Figure 4C). The tremoring phenotype and impaired motility might have contributed to their poor weight gain despite our attempts to compensate for impaired movement by making food readily accessible by placing moistened chow and gel food on the cage floor.

### DigiGait analysis of impaired motor function in Hup23 mice

We examined Hup23 mice using the DigiGait Imaging System in order to detect and quantify impairment in coordination and motor function in transgenic animals. The DigiGait system uses high-speed videography to image the underside of mice as they walk on a motorized transparent treadmill belt. Adult mice move at velocities of 14 - 43 cm/sec during spontaneous walk/trot locomotion [18]. The Hup23 mice in each line and their Ntg littermates were examined at walking speeds that ranged from 2 - 20 cm/sec as outlined under Material and Methods starting at P28 (n = 10) or P20 [for line 43; n = 4]. The trials were performed every other day until the mice were unable to run on the treadmill. In each transgenic line, there was a clear difference between Hup23 mice and Ntg littermates in all the parameters, including stride length and frequency. Before the onset of overt symptoms, transgenic mice already showed difficulty maintaining their speed and occasionally galloped by moving the hind limbs synchronously. Additional movie files illustrate these differences [see Additional files 3 and 4]. Because the motor deficits were so striking when the treadmill was held at 20 cm/sec, we resorted to performing trials at progressively lower treadmill speeds whenever the mice had trouble running at a given speed, and noted the speed at which the animals were able to comfortably run for 5 to 10 seconds during the trial. In hundreds of trials Ntg littermates regardless of age, gender, or which line they were from, had no trouble running with the treadmill held at 20 cm/sec. Similarly, Hup23 mice were able to run at the treadmill speed of 20 cm/sec at P24 and P28. However, they progressively lost the ability to reach and keep up with the treadmill speed (Figure 4D; [see Additional file 5]). Statistical analysis (one-sample *t*-test) revealed significant age-associated decline of running speed in Hup23 mice by P32 (*p *< 0.01 on P32; *p *< 0.0001 at all later time points).

Analyzing Hup23 mice using the DigiGait Imaging System, which measures running and motor activity parameters with high accuracy, revealed defects in motor function before visible signs of tremor or other symptoms related to movement became apparent. For instance, subtle symptoms in movement were visually observed in Hup23 line 06 at P37 whereas the motor deficits were readily observed on DigiGait trial one week earlier, at P30. A comparison of the level of p23 overexpression and the age when Hup23 animals exhibited differences in DigiGait treadmill performance is summarized in Table 1. In general, the higher the p23 expression level the earlier the motor dysfunction was detected (Table 1).

### Normal neuronal morphology in Hup23 mice

**Figure 5:**
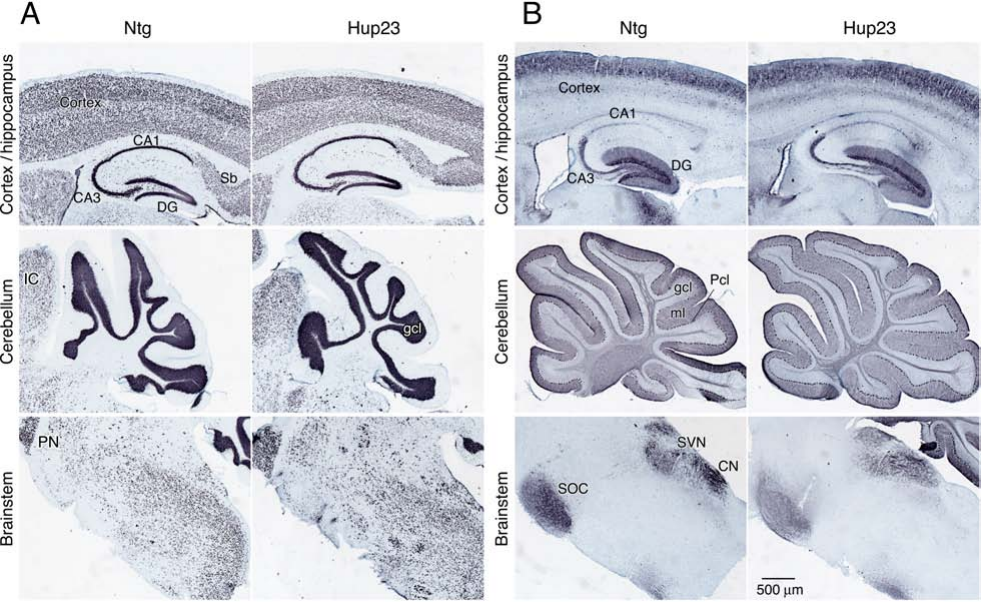
**Immunohistochemical analysis of neuronal markers in Hup23 mice**. Sagittal brain sections of Hup23 and Ntg brain were processed by immunoperoxidase staining with antibodies against NeuN (A) and calbindin (B). Images of cortex and hippocampus, cerebellum, and brainstem are shown. The following areas are indicated: *DG*, dentate gyrus; *Sb*, subiculum; *IC*, inferior culliculus, *PN*, pontine nuclei, *gcl*, granule cell layer; *ml*, molecular layer; *Pcl*, Purkinje cell layer; *DCN*, deep cerebellar nuclei, *SOC*, superior olivary complex, *SVN*, spinal vestibular nucleus, *CN*, cuneate nucleus. Note that the overall immunoreactivity for both markers is comparable in Hup23 and Ntg brain.

To examine the neuronal morphology in Hup23 mice we performed immunostaining using neuron-specific markers: neuronal nuclear antigen (NeuN) to stain all neuronal cells and calbindin D-28K to stain select neuronal populations in the brain including Purkinje cells. Immunohistochemical staining using antibodies against NeuN failed to reveal marked differences in staining intensity or the distribution of NeuN-positive immunoreactivity in cortex, hippocampus, cerebellum and brain stem (Figure 5A). Similarly, Hup23 and Ntg littermates revealed no marked difference in calbindin staining in outer layers in cortex, hippocampal mossy fiber terminals, cell bodies in the granule cell layer and dendrites that extended into the molecular layer of dentate gyrus, Purkinje cells and their dendrites in cerebellum, and staining along fibers and termini within vestibular nuclear complex, pontine gray and superior olivary complex in brainstem (Figure 5B). Moreover, TUNEL staining failed to reveal signs of escalated level of cell death in mouse brain upon overexpression of p23 in Hup23 mice (data not shown).

**Figure 6:**
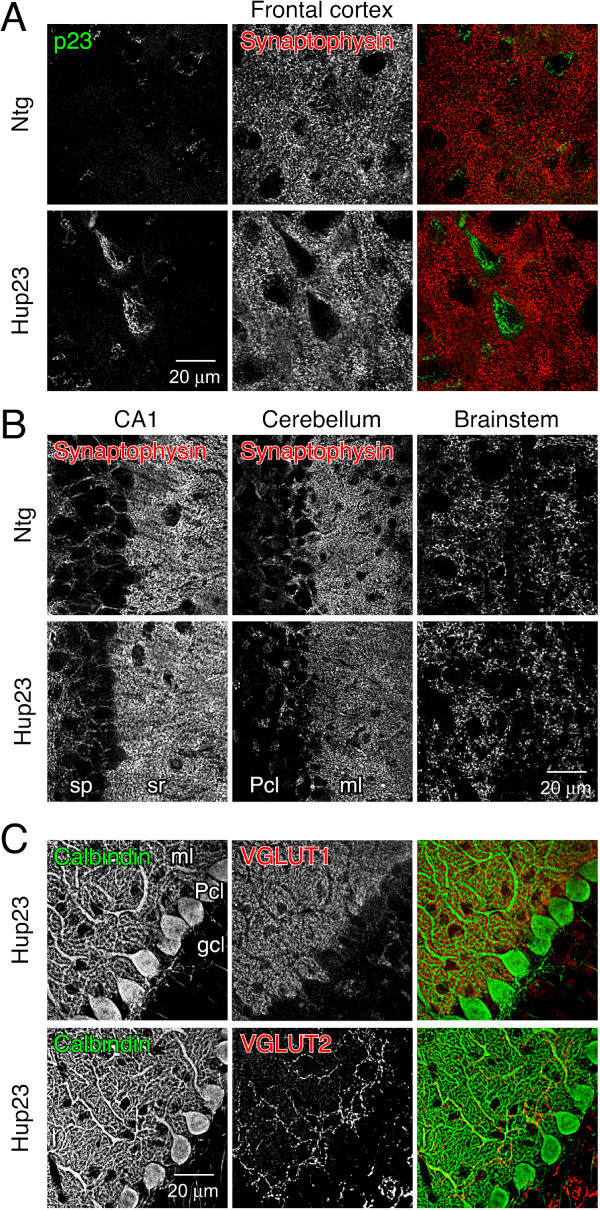
**Analysis of p23 and synaptic marker expression**. Double immunofluorescence analysis was performed on sagittal brain sections stained with indicated antibodies using confocal microscopy. ***A***, Pyramidal neurons in frontal cortex of Hup23 mice show overexpression of p23 (green) and similar presynaptic distribution of synaptophysin (red). ***B***, Synaptophysin staining in hippocampal CA1 region, cerebellum, and brainstem appears indistinguishable between Hup23 and Ntg mice. ***C***, Normal appearance of Purkinje cells visualized by calbindin staining, as well as VGLUT1 and VGLUT-2 labeling of parallel fibers and climbing fibers, respectively, in cerebellum of Hup23 animals. The following areas are indicated: *Pcl*, Purkinje cell layer; *gcl*, granule cell layer, *ml*, molecular layer; *sp*, stratum pyramidale; *sr*, stratum radiatum.

**Figure 7:**
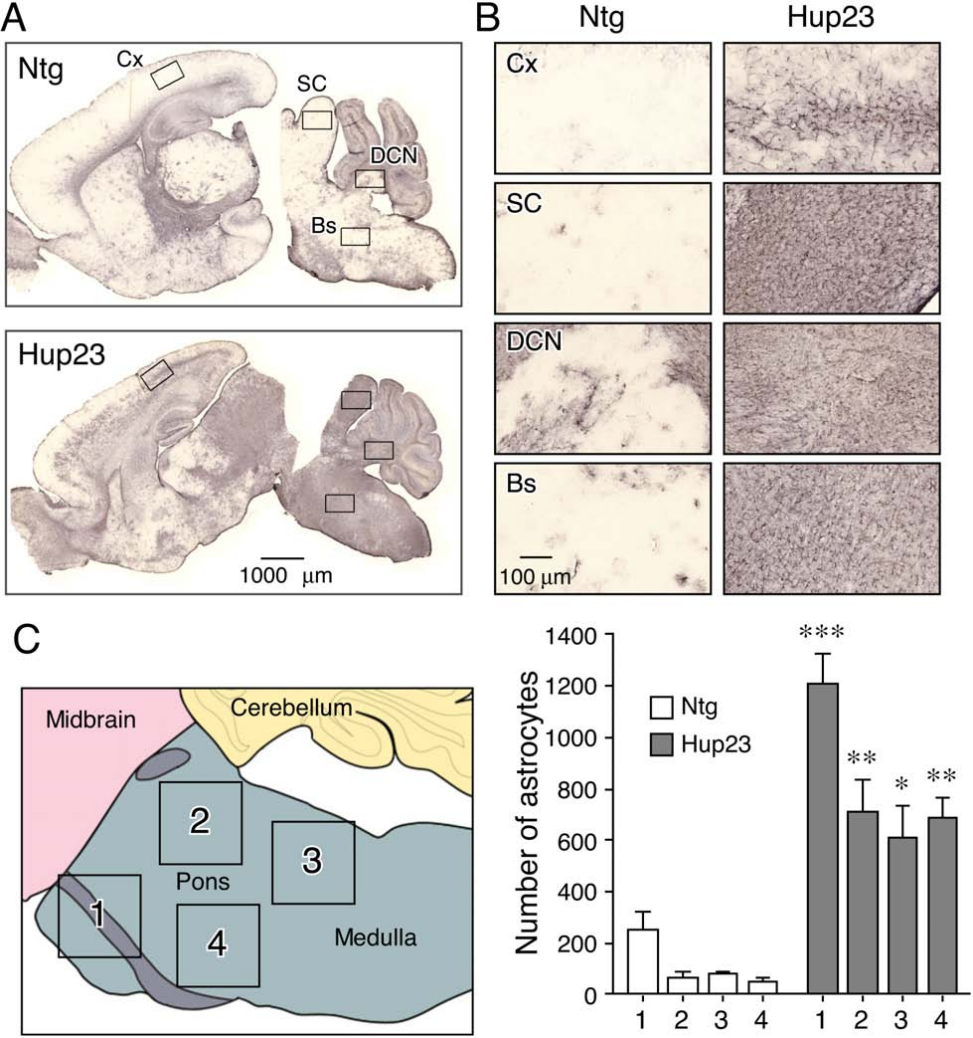
**Gliosis in Hup23 Tg mouse brain**. ***A***, Sagittal brain sections of Hup23 Tg mice and their Ntg littermates were stained with an antibody against GFAP. A pair of representative images is shown at lower magnification. ***B***, Higher magnification images of cortex (Cx), superior colliculus (SC), deep cerebellar nuclei (DCN), and brainstem (Bs) [regions indicated by boxes in panel A] are shown. ***C***, Quantification of astrogliosis in Hup23 brainstem. The number of star-shaped GFAP positive astrocytes was counted in four defined regions within brainstem (see the schematics on the left) in Hup23 mice and littermates from 4 different lines, and the mean ± SEM is plotted. Pair wise *t*-test ***, *p *< 0.001, ** *p *< 0.01, * *p *< 0.05.

To further characterize neuronal morphology in Hup23 mice, we performed double-immunofluorescence staining of p23 and presynaptic marker synaptophysin, dendritic marker microtubule-associated protein 2 (MAP2), or myelinated axon bundle marker myelin basic protein (MBP). Confocal microscopy analysis revealed p23 overexpression throughout the cortex, especially obvious in large pyramidal neurons (Figure 6A). No discernable difference in the staining pattern or intensity of presynaptic marker synaptophysin was observed in the vicinity of neurons overexpressing p23 (Figure 6A). Moreover, similar presynaptic staining was observed in hippocampal CA1 region, cerebellar Purkinje cell and molecular layers, and brainstem (Figure 6B), despite overall higher p23 expression in Hup23 mice (see Figure 7). MAP2 staining also revealed no marked difference between Hup23 and Ntg littermates (not shown).

Since Hup23 mice developed tremor and symptoms similar to those caused by cerebellar ataxia, we decided to further characterize Purkinje neurons in cerebellum. Presynaptic vesicular glutamate transporters (VGLUTs) are responsible for transport of L-glutamate, the predominant excitatory neurotransmitter in the central nervous system into the synaptic vesicles, and consequently VGLUT1 and VGLUT2 are used as markers of excitatory presynaptic terminals [19]. VGLUT1-deficient mice exhibit typical neurological phenotypes including uncoordinated movement [20], and alterations in VGLUT1 and VGLUT2 expression level have been suggested to account for the motor deficits observed in Parkinson's disease [21]. In this context, we examined by double immunofluorescence staining the distribution of VGLUT1 or VGLUT2 along with calbindin in cerebellum of Hup23 mice. This analysis revealed normal appearance of calbindin staining in the soma and dendrites of Purkinje neurons, and the expected pattern of VGLUT1 and VGLUT2 staining in the two excitatory afferents, with VGLUT1 being localized in parallel fibers (red, upper panel) and VGLUT2 in dendrites of Purkinje cells and climbing fibers (red, lower panel) (Figure 6C and Additional file 6). Thus, overexpression of p23 does not alter synaptic wiring in cerebellar Purkinje cells and the distribution of VGLUT1 and VGLUT2 in cerebellar excitatory afferents of Hup23 Tg mice.

### Glial cell activation and defective myelination in brainstem of Hup23 mice

**Figure 8:**
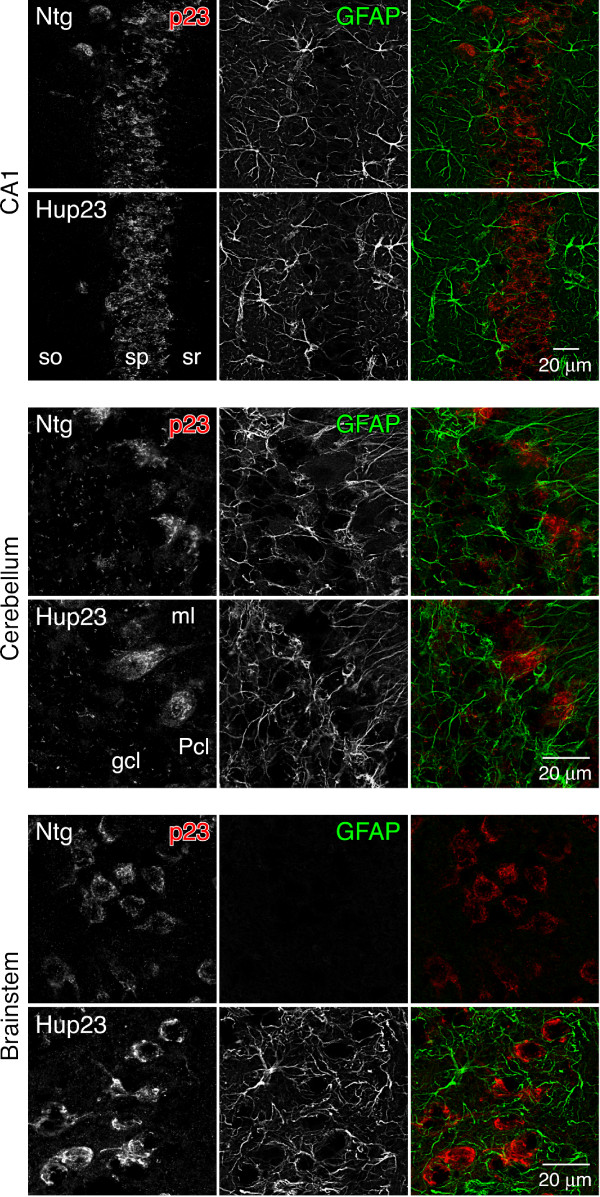
**Analysis of p23 and GFAP expression in p23 mice**. Double immunofluorescence analysis was performed on sagittal brain sections stained with antibodies against p23 (red) and GFAP (green) using confocal microscopy. GFAP staining in hippocampal CA1 region and cerebellum appears indistinguishable between Hup23 and Ntg mice. However, marked gliosis is apparent in brainstem. Overexpression of p23 is observed in all three areas.

Since neuronal cells in Hup23 mouse brain seemed to be normal (i.e. both the number and the morphology of neuronal cells were not markedly altered), we decided to stain brain sections with antibodies against non-neuronal cells. We first examined astrocytes, the most abundant cell type in the brain. In response to a spectrum of brain injury conditions ranging from acute trauma or stroke to inflammatory conditions and neurodegeneration, astrocytes become hypertrophic and markedly upregulate expression of the intermediate filament protein GFAP, a process referred to as 'astrogliosis' [reviewed by [22]]. When we stained brain sections of Hup23 mice with GFAP antibody, prominent activation of astrocytes was observed consistently in brainstem, inferior colliculus and deep cerebellar nuclei in each of the six Hup23 lines (Figure 7A and 7B). In addition, two high expressing Hup23 lines, 43 and 61, showed activation of astrocytes in cortex (Figure 7A). Confocal microscopy analysis of sections stained with p23 and GFAP antibodies revealed neuronal overexpression of p23 along with robust activation of astrocytes in brainstem (Figure 8). In contrast, GFAP staining in CA1 region of hippocampus and cerebellum was comparable between Hup23 and Ntg mice in 4 of the 6 Hup23 lines (lines 06, 74, 76, and 89) (Figure 8). Quantitative analysis of mice from 4 lines (lines 06, 43, 74 and 89) showed significant increase in the number of GFAP-positive astrocytes in brainstem of Hup23 mice as compared to Ntg littermates (Figure 7C).

**Figure 9:**
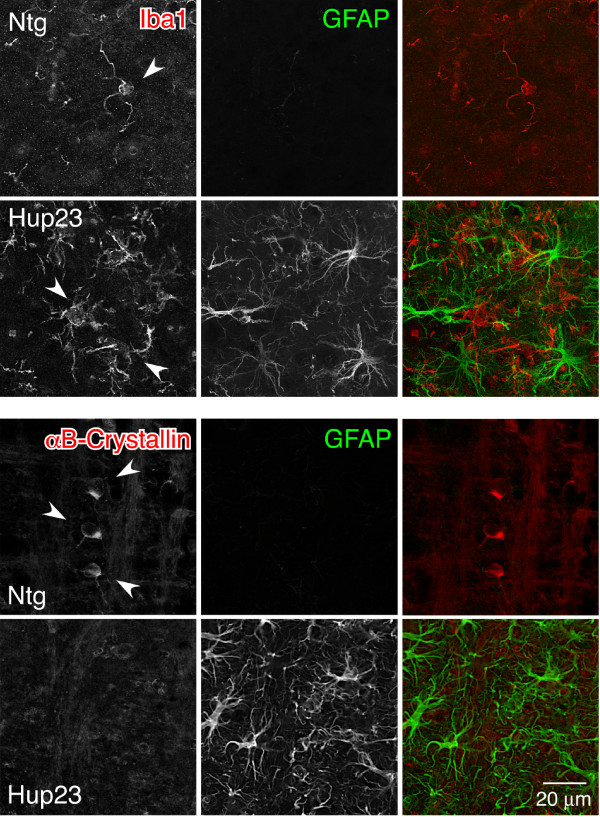
**Analysis of microglia and oligodendrocytes in Hup23 mice**. Double immunofluorescence analysis was performed on sagittal brain sections stained with antibodies against GFAP (green) and microglial marker Iba1 or αB-crystallin, which stains oligodendrocytes in brain (red), using confocal microscopy. Hup23 mice show robust microglial activation but weak αB-crystallin labeling of oligodendrocytes.

The above results are consistent with neuronal p23 overexpression causing certain perturbation of brainstem where the highest level of overexpression was observed. Next, we performed immunofluorescence analysis of microglia and oligodendrocytes in brains of Hup23 mice. When the nervous system senses an injury or signs of neuronal dysfunction, it triggers a rapid cellular response resulting in neuroinflammation mediated by microglia [23]. Resting microglia are characterized by small rod-shaped cell body and thin ramified processes originating from the cell body. The activation of microglial cells in pathological conditions is manifested primarily by their proliferation. Moreover, during the multistate activation process microglia undergo a series of changes whereby their cell bodies become rounder and the processes become thicker [24,25]. When we stained microglia with an antibody against Iba1, a marker that recognizes both resting and activated microglia, we found that microglia in Hup23 mouse brainstem had typical amoeboid morphology of activated microglia whereas the microglia in Ntg mouse brainstem were at resting state (Figure 9). In contrast to astrocytes and microglia, which proliferate in response to injury or neuronal dysfunction, oligodendrocytes are vulnerable to neuronal injury such as inflammation and oxidative stress. αB-crystallin, a member of the class of small heat shock proteins, is known to be expressed in oligodendrocytes and upregulated under certain pathological conditions [26]. When we performed double immunofluorescence staining with antibodies against αB-crystallin and GFAP, we observed αB-crystallin staining of oligodendrocytes in brainstem of Ntg mice but not in Hup23 mice, despite intense activation of astrocytes (Figure 9). Similar to what we observed with GFAP staining, Iba1 and αB-crystallin staining in cortex and hippocampus were also similar between Ntg and Hup23 mice.

**Figure 10:**
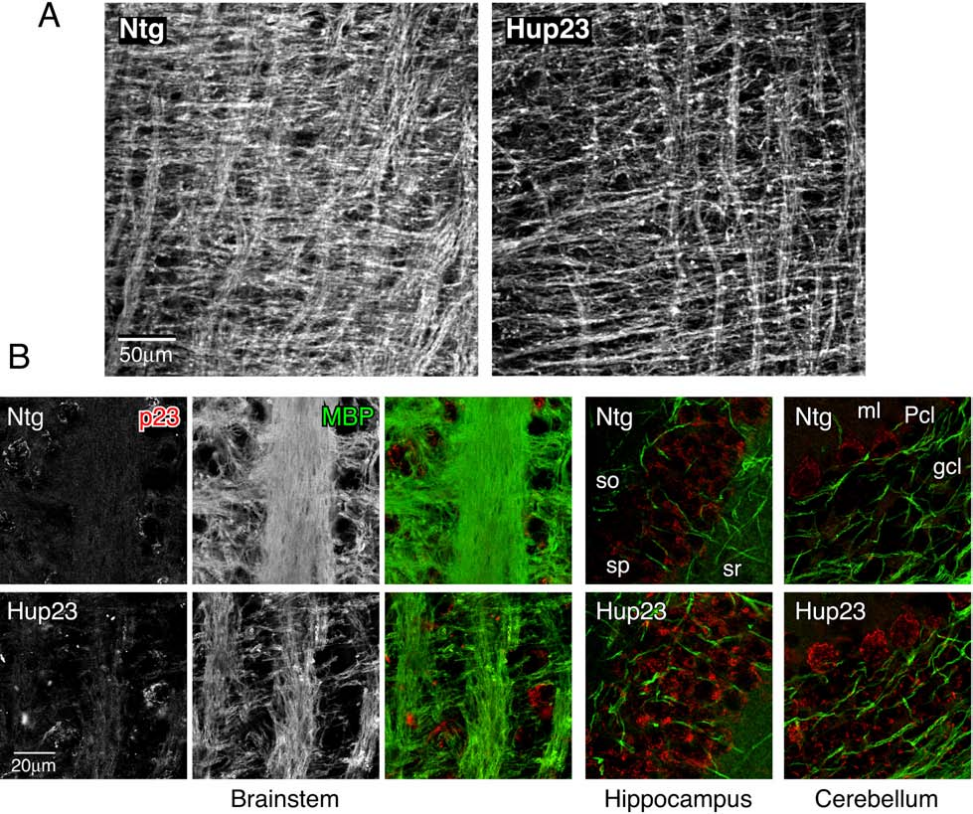
**Abnormal MBP staining in brainstem of Hup23 mice**. Double immunofluorescence analysis was performed on sagittal brain sections stained with antibodies against p23 (red) and MBP (green) using confocal microscopy. ***A***, Representative lower magnification images of brainstem reveal thinner myelinated bundles in Hup23 brain. ***B***, Higher magnification images of brainstem, staining in hippocampal CA1 region and cerebellum are shown. Unlike the apparent difference observed in brainstem, MBP staining in hippocampus and cerebellum is comparable between Hup23 mice and Ntg littermates.

Finally, we stained brains sections with an antibody against MBP, a major component of myelin and found that staining intensity and pattern of staining were comparable between Hup23 and Ntg littermates in cortex, hippocampus and cerebellum (Figure 10B and not shown). In contrast, we observed sparse MBP staining in brainstem at lower magnification (Figure 10A). Confocal microscopy at higher magnification clearly shows the presence of thinner myelinated bundles in brainstem of Hup23 mice as compared with larger bundles of myelinated axons observed in Ntg mice (Figure 10B). This difference between Hup23 mice and littermates in MBP staining, which is indicative of defective myelin formation, was observed in brainstems of all Hup23 lines examined. Thus, in brainstems of Hup23 mice astrocytes and microglia are activated whereas myelin formation is partially impaired in response to neuronal overexpression of p23. Unfortunately, at the time when we discovered this phenotype we were unable to generate any additional F1 offspring in order to further characterize hypomyelination or loss of myelinated axons by performing ultrastructural analysis of myelin in Hup23 mice.

### p23 overexpression causes Golgi fragmentation

**Figure 11:**
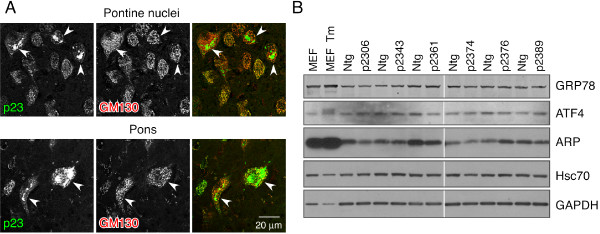
**Golgi fragmentation in Hup23 brainstem**. ***A***, Double immunofluorescence analysis of Hup23 brains stained with antibodies against p23 and GM130. Confocal images of neurons in pontine nuclei (upper panels) and a region in the middle of brainstem (lower panels) are shown. *Arrowheads *indicate abnormal p23 localization and Golgi fragmentation (visualized by GM130 staining) in neurons expressing high levels of p23. ***B***, Immunoblot analysis of UPR markers in brainstem of Hup23 mice. Seventy five μg of total protein lysates of brainstem of Hup23 and non-transgenic littermates were fractionated on 4-20% SDS-PAGE gels and immunoblotted using antibodies against the indicated proteins. Hsc70 and GAPDH levels served as loading controls. Lysates from mouse embryonic fibroblasts (MEF) exposed overnight to 2 μg/ml tunicamycin (Tm) were analyzed on the same gels as positive control for the activation of the UPR.

Previously it was reported that overexpression of p23 in cultured proliferating cells results in fragmentation of the Golgi apparatus and expansion of smooth ER membranes in a subset of transfected cells [6]. To determine whether p23 overexpression altered Golgi architecture in post-mitotic neurons in brain, we performed double immunofluorescence labeling with antibodies against p23 and GM130, a cis-Golgi marker. By confocal microscopy, we observed normal Golgi apparatus morphology and near complete overlap between p23 and GM130 localization in p23 overexpressing pyramidal neurons located within the cortex and Purkinje neurons in cerebellum (not shown). However, in brainstem we found a subset of neurons located in pontine nuclei and in the center of pons that contained abnormal p23-positive structures, which were not labeled by GM130 antibody (Figure 11A). In these neurons the Golgi apparatus has lost its characteristic continuous ribbon morphology and appeared to have undergone fragmentation (Figure 11A).

It is well known that p24 family proteins participate in bidirectional transport of proteins between the ER and Golgi, and facilitate ER quality control [3,4,6,7,27]. Moreover, loss of p24 function in yeast causes activation of the UPR [28]. Therefore, we considered the possibility that overexpression of p23 might cause protein misfolding and evoke the UPR. To this end, we performed real-time PCR analysis of mRNA isolated from Hup23 and Ntg littermate in lines 06, 43, 61, and 74 to assess the expression of proapoptotic transcription factor CHOP, a well-characterized target of the mammalian UPR. We found upregulation of CHOP expression in lines 43 and 61 (the lines with the highest level of Hup23 expression), but not in lines 06 and 74 (the lines with low levels of Hup23 expression) (data not shown). To extend this observation, we performed immunoblot analysis of ER chaperone GRP78, a well-known marker of the UPR. In addition, we examined activating transcription factor ATF4, a protein whose steady-state levels are very low under normal conditions. In cells experiencing ER stress, RNA-activated protein kinase-like endoplasmic reticulum kinase PERK induces phosphorylation of eukaryotic translation initiation factor eIF2α, which facilitates enhanced translation of *Atf4 *mRNA through internal ribosomal entry. ATF4 then activates the upregulation of a subset of UPR-target genes through a transcriptional cascade involving ATF3 and CHOP [29,30]. Finally, we examined the levels of ER-resident luminal protein ARP (also called ARMET), which is widely expressed in the nervous system and upregulated in neurons by ER stress [31-33]. Immunoblot analysis of brainstem lysates from all six lines of Hup23 mice revealed little sign of increased accumulation of these three UPR-associated proteins (Figure 11B). Collectively, the results above indicate that high level p23 overexpression leads to mislocalization of p23 and Golgi fragmentation in a subset of neurons in the brainstem, but these defects do not lead to overt activation of the UPR.

## Discussion

We report here the generation and characterization of transgenic mice overexpressing human p23 in neurons throughout the central nervous system, under the control of *Thy-1.2 *promoter. We found that neuronal overexpression of p23 in mice causes a complex set of neurological problems with onset in the first few weeks after birth, progressive motor deficits, post-natal growth retardation, infertility, and premature death. Despite this morbid phenotype, the major brain structures appeared normal in Hup23 mice, and neurons throughout the brain and spinal cord had normal morphology when analyzed by staining with neuronal markers and showed no overt sign of enhanced apoptosis. However, we observed striking astrogliosis, activation of microglia and defects in myelination in brainstem of Hup23 mice. Furthermore, p23 overexpression led to increased burden of protein misfolding in the ER as evidenced by the activation of integrated stress response in brainstem. These results suggest that p23 function in the early secretory pathway is critical for neuronal function and even modest overexpression of this protein is sufficient to cause deleterious cellular dysfunction, which ultimately manifests as a complex neurological phenotype in transgenic mice.

p24 family proteins exist and function as stable oligomeric complexes, and the stability of each p24 protein depends on successful heteromeric assembly with other family members. For example, p23 can exist as monomers, homodimers, and heterodimers with p24, p25, and p27, but not p26 [34,35]. Knockdown of p23 expression reduces steady-state levels of p24, p25, p27, and tp24, supporting their existence as heteromers and their dependency on each other to form stable complexes [8,36-38]. Our findings that even modest neuronal overexpression of p23 (~1.4 fold endogenous levels) elicits a range of phenotype in transgenic mice, and the severity of the phenotype correlated well with the level of p23 overexpression, suggest that a delicate balance exists in the relative abundance of p24 proteins in neurons. In contrast, mice with targeted deletion of one allele of the gene encoding p23, which resulted in 70% loss of steady-state p23 expression, were viable and free of any neurological deficits [8]. Notably, the steady-state levels of other p23 family proteins were concomitantly reduced in heterozygous p23 animals [8]. However, in Hup23 mice there was virtually no compensatory upregulation of p24 following overexpression of p23, even when p23 levels were elevated more than 10-fold in the brainstem of line 61 (Figure 2). This observation is in agreement with lack of changes in the steady-state levels of endogenous p24 proteins following transgenic expression of p24δ1 (p23 orthologue) under the control of the proopiomelanocortin promoter in the melanotrope cells of the intermediate pituitary [39]. Therefore, it is possible that the relative ratio of different p24 family proteins in certain cell types, such as post-mitotic neurons, is more critical than the absolute level of individual p24 proteins.

Previous studies have shown that at least two p24 family proteins are essential for mouse embryonic development: p23 knockout embryos die at E4.5 prior to implantation, and p24 knock out embryos die at E10.5 [8,40]. In either case, expression of other p24 family proteins was severely compromised, in agreement with the notion that steady-state levels of p24 proteins are interdependent and complex formation regulates their stability. While it has not been firmly established, it is very likely that lethality of p23 and p24 knockout embryos is the consequence of abnormal protein trafficking due to loss of function of p24 family proteins during early embryogenesis. In this regard, the *Thy-1.2 *promoter used to generate Hup23 mice is known to drive transgene expression in subsets of neurons as early as E13, although robust neuronal expression is consistently observed shortly after birth [15,17]. Our observation that Hup23 mice are normal at birth and showed no symptoms for the first two weeks after birth indicates that neuronal overexpression of p23 is not deleterious during late stages of embryonic and early post-natal development (Figure 4B). This agrees well with our previous observations that the steady-state level of endogenous p23 in mouse brain is high during embryonic development and on P0, but declines by 50% in the first four weeks after birth [14]. High-level p23 expression during embryogenesis and the time around birth may also be an indication of a higher demand of select trafficking facilitated by p23 at early stages in life. However, sustained high level neuronal p23 expression during post-natal development in Hup23 mice, especially at time when endogenous p23 expression begins to decline [14], was deleterious to neuronal functions as evidenced by the complex neurological phenotype observed in Hup23 mice.

Confocal microscopy analysis confirmed that p23 is overexpressed only in neurons in Hup23 mice; proliferating astrocytes and activated microglia do not show evidence of p23 overexpression (Figures 6 and 8). Consistent with immunoblot analyses where we quantified higher steady-state levels of p23 in brainstem of multiple Hup23 lines, we observed overall higher p23 staining in neurons in brainstem as compared with pyramidal neurons in cortex and hippocampus. Moreover, while overexpressed p23 localized to Golgi apparatus in pyramidal neurons in cortex and hippocampus as well as Purkinje neurons in cerebellum, a subset of neurons in brainstem showed p23 staining as clusters that were not labeled by the cis-Golgi marker GM130 (Figure 11). This later observation is highly reminiscent of mislocalization of overexpressed p23 in expanded ER membranes in cultured cells [5,6,10]. We believe that clustering of p23 in neurons localized in brainstem is simply an outcome of the overall higher p23 expression in this brain region. Consequently, we observed adverse cellular reactions more readily in brainstem, such as extensive astrocyte proliferation, microglial activation and partial loss of myelination. How neuronal overexpression instigates these complex cellular reactions is an interesting question. Nevertheless, premature death and the inability to generate Hup23 F2 offspring curtailed our investigation. Mapping with greater precision the neuronal population responsible of the phenotype will require the generation of additional mouse models to achieve p23 overexpression in a Cre-dependent manner by crossing transgenic mice harboring "floxed" p23 expression constructs with specific driver lines (such as the HB9^cre ^line to direct p23 overexpression selectively in motoneurons).

While the effect of p23 on protein trafficking has been primarily investigated in cells where endogenous p23 expression has been silenced using siRNA, overexpression of p23 in culture cells has also shown to affect protein trafficking [6,10]. Since overexpression of p23 displaces endogenous p23 from cis-Golgi into clustered smooth ER membranes, it is likely that normal p23 function in protein trafficking is compromised in cells upon p23 overexpression. Accumulation of misfolded protein is a common feature in several neurodegenerative diseases including Huntington's disease, which is characterized by motor abnormalities [41]. Furthermore, ER stress has been implicated in pathogenesis of various myelin disorders, including Charcot-Marie-Tooth disease and multiple sclerosis [42]. Therefore we examined whether UPR is activated following high-level p23 expression in Hup23 animals. Although real-time PCR analysis showed activation of CHOP expression in two lines, immunoblot analysis of all six lines failed to demonstrate steady-state accumulation of UPR associated proteins such as GRP78, ATF4 and ARP. Precisely what effect p23 overexpression has on the neuronal early secretory pathway remains to be investigated. Nevertheless, results of our analysis clearly shows that the complex phenotype observed in Hup23 mice can not be attributed to accumulation of misfolded proteins to the extent that it triggers robust UPR activation.

## Conclusions

Our characterization of neurological phenotype and premature death in Hup23 mice has broader implications for AD research. Diminution of p23 expression in cultured cell lines by siRNA increases AD-associated Aβ production, through mechanisms that involve APP trafficking and γ-secretase processing of APP-derived C-terminal fragments [11,37]. Progressive decline in p23 expression one month after birth in mice and lower steady-state p23 levels in individuals with AD [14] prompted us to suggest that lower levels of p23 expression might be a critical factor that modulates the extent of Aβ production, and consequently contribute to disease pathogenesis. Based on negative regulation of Aβ levels by p23 expression and physiological decline in p23 expression during aging, it is particularly interesting to consider increasing p23 expression or enhancing p23 as a potential strategy to reduce cerebral Aβ levels. However, as evidenced by the phenotype of Hup23 mice, pan-neuronal expression has serious consequences. Still, it remains to be determined whether maintaining high p23 expression in select neuronal populations, such as neurons in the forebrain, will attenuate Aβ pathology in mouse models of amyloid deposition while circumventing the phenotypes such as tremor and uncoordinated movements observed in Hup23 mice. Results of the present investigation will be highly instructive for such future efforts.

## Methods

### Generation of p23 transgenic mice

The cDNA expressing human p23 was subcloned into the *Thy-1.2 *genomic expression vector provided by Dr. P. Caroni (University of Basel, Basel, Switzerland) [15]. The *Thy-1.2*-Hup23 expression cassette was released by digestion with *EcoR*I and *Pvu*I, gel-purified, and injected into C57BL/6 fertilized oocytes at the Biologicl Sciences Division Transgenics/ES Cell Technology Mouse Core Facility at the University of Chicago. Ninety-three potential founders were generated and screened for the presence of the transgene by PCR using the following primers: p23F2702 (5'-ATTGGTCCTTGCCATCTCCTTCCA) and p23R3168 (5'-TTGTTGACTCGTTGGTATCACGCA) corresponding to sequences within human p23 cDNA. Control reactions were performed using primers Tmed10F (5'-GGAGGATGAAGAACTGAAGGTCG) and Tmed10R (5-TGTTTCTTGGAGGGGCATAGC) to amplify a 712-bp segment of the endogenous *Tmed10 *locus. Founders were bred to C57BL/6J animals and all experiments described here were performed on F1 offspring. Body weights of the mice were measured biweekly for line 06, 43, 74, and 76. The weights of littermates of the same sex were used to calculate the % difference in body weight over time. The differences in body weight between Hup23 and Ntg littermates on the last day of measurement were analyzed by one-way ANOVA and Tukey post-hoc comparison.

All experiments involving animals were performed with prior approval from the University of Chicago Institutional Animal Care and Use Committee in accordance with federal regulations issued by the United States Department of Agriculture, the National Institutes of Health Office of Laboratory Animal Welfare, and the Association for the Assessment and Accreditation of Laboratory Animal Care International.

### DigiGait analysis

The DigiGait trials were performed on a total of 14 Hup23 mice and 11 Ntg littermates every other day using DigiGait™ Imaging System (Mouse Specifics, Inc.) until the Hup23 mice were not able to run or died between trials. At their first trials, mice were trained to run on the treadmill by gradually increasing the speed of the treadmill from 2 to 5, 10, and eventually 20 cm/sec. In a test trial, a mouse ran in the treadmill at a certain speed for about 5 seconds. If a mouse can keep up with the treadmill, the speed of the treadmill was recorded as the running speed of the mouse. If a mouse failed to gain speed and run, the trial was repeated after 5 min with the treadmill held at the next lower speed. All Ntg mice were able to run at the speed of 20 cm/sec at all ages tested. Therefore, a one-sample *t*-test was performed to calculate statistical difference between the treadmill speeds of Hup23 and Ntg mice.

### Antibodies

Rabbit polyclonal p23 antisera against mouse p23 (residues 32-57) and flotillin-2, and guinea pig GRP78 antibody have been described [37,43,44]. Polyclonal p24 antiserum was raised against a glutathione *S*-transferase fusion protein corresponding to residues 30-201 of human p24A. Polyclonal ARP antiserum was raised against a synthetic peptide corresponding to the C-terminal 29 amino acids of mouse ARP. The following commercially available antibodies were used in this study - mAb: synaptophysin, MAP2 and GFAP (Sigma), NeuN (Chemicon), MBP (Covance), GM130 (BD Transduction Laboratories), ATF4 and Hsc70 (Santa Cruz Biotechnology), GAPDH (Abcam); polyclonal antibodies: Calbindin-D-28K (Sigma), VGLUT1 and VGLUT2 (Millipore), GFAP (Dako), Iba1 (Wako), and anti αB-crystallin (Serotec).

### Immunohistochemistry and immunofluorescence staining

Mice were anesthetized by isoflurane inhalation before perfusion with ice-cold PBS. Mouse brains and spinal cords were harvested and kept on ice. Brains were cut midsagittally, and one half was further dissected into forebrain, brainstem, and cerebellum and stored at -80°C for use in Western blot analysis. The other half of brain and spinal cord were immersion fixed in 4% paraformaldehyde for 48 h and cryoprotected in PBS containing 30% sucrose. Thirty μm-thick sections were cut on a cryostat and processed following a free-floating procedure for either enzyme-linked immunoperoxidase or double-immunofluorescence staining essentially as described previously [37]. Immunoperoxidase stained sections were examined under a Zeiss Axioplan 2 microscope and images were acquired using an AxioCam color camera (Carl Zeiss). Alternatively, stained sections were imaged on a CRi Pannoramic Scan Whole Slide Scanner (Cambridge Research and Instrumentation) using 40 × 0.95 NA LWD Zeiss objective and Allied Vision Technologies Stingray F146C, 4.6 μm pixel size color camera. Tiled images were analyzed using 3DHistech software (Cambridge Research and Instrumentation). Images of fluorescence staining were acquired on a Leica SP5 Confocal microscope using 10 × (0.4 NA), 20 × (0.7 NA), 63 × (1.4 NA), or 100 × (1.46 NA) objectives and processed using ImageJ software [45]. Identical settings were used between Ntg and Hup23 samples for image acquisition and processing.

### Western blot analysis

Detergent lysates of dissected brain regions and spinal cord were prepared as described previously [46]. Fifteen or ninety μg of total protein were separated on 4-20% SDS-PAGE gels and transferred to Immobilon-FL transfer membranes (Millipore). The membranes were blocked, incubated in primary antibodies for 2 h, and IRDye 800CW donkey anti-rabbit IgG (LI-COR Biosciences) for 1 h, and scanned using Odyssey Imager (LI-COR Biosciences). The blots were probed with p23, p24, and flotillin-2 antibodies. p23 signal intensities were normalized to flotillin-2 signals. Two-fold dilutions of line 61 brain lysates were used to ensure accurate quantification of signal intensities. Seventy five μg of total protein were used for the analysis of UPR markers and the blots were developed using chemiluminescence.

### RT-PCR and real-time PCR

Total RNA was isolated from homogenized brainstem using Trizol reagent (Invitrogen) and RNeasy Mini Kit (Qiagen). cDNA was synthesized using SuperScript III First-Strand Synthesis System (Invitrogen). Real-Time PCR was performed using SYBR GreenER qPCR SuperMix (Invitrogen) on iCycler iQ Real-Time PCR Detection System (BIO-RAD). Melting curve analysis was used to identify the PCR products and Efficiency^ΔΔCt ^method was used to quantify the relative abundance of *Ddit3 *mRNA using *Gapdh *mRNA levels as control [47]. The primers used are as follows: *Ddit3 *(CHOP) 5' GTCCAGCTGGGAGCTGGAAG and 5' CTGGTCAGGCGCTCGATTTCC; *Gapdh *5' GATGACATCAAGAAGGTGGTGAAG and 5' GTGAGGGAGATGCTCAGTGTTGG.

## List of abbreviations

Hup23 mice: human p23 transgenic mice; COPI: coated protein complex I; ER: endoplasmic reticulum; AD: Alzheimer's disease; APP: amyloid precursor protein; Aβ: β-amyloid; UPR: unfolded protein response; Ntg: non-transgenic; NeuN: neuronal nuclear antigen; MAP2: microtubule-associated protein 2; MBP: myelin basic protein; VGLUT: vesicular glutamate transporter.

## Competing interests

The authors declare that they have no competing interests.

## Authors' contributions

PG, JR, CGF, KSV and ATP designed experiments, performed the molecular, immunohistochemical, and behavioral characterization of p23 transgenic mice, and participated in data analysis. PG, VPB, SK, and GT participated in image acquisition and analysis of immunostaining data. LAZ participated in characterizing the disease phenotype. PG and GT wrote the manuscript. SK, LAZ, and ATP helped to draft the manuscript. GT conceived of the study, designed experiments, coordinated data analysis, and prepared the final manuscript. All authors read and approved the final manuscript.

## Supplementary Material

Additional file 1 **Neurological defects in Hup23 mice from 6 transgenic lines**. Hup23 mice from 6 transgenic lines display similar neurological defects with different levels of severity depending on the level of transgene expression. The videos were recorded on the days indicated in each panel. Click here for file

Additional file 2 **Side view of Hup23 line 43 mouse on a stationary DigiGait treadmill**. A closer view of Hup23 mouse from line 43 displaying the complex neurological defects (right) and its Ntg littermate (left). The videos were recorded on P30 when the animals were placed on a stationary DigiGait treadmill. Click here for file

Additional file 3 **Side view of Hup23 line 43 mouse running on DigiGait treadmill**. Side view of Hup23 mouse from line 43 and Ntg littermate running on the DigiGait treadmill held at the speed of 20 cm/sec. The videos were recorded on P30. Click here for file

Additional file 4 **Bottom view of Hup23 line 74 mouse running on DigiGait treadmill**. Bottom view of a Hup23 mouse from line 74 and Ntg littermate running on the DigiGait treadmill held at the speed of 20 cm/sec. The videos were recorded on P30. Click here for file

Additional file 5 **Rapid decline of Hup23 line 74 mouse performance on DigiGait treadmill**. The same Hup23 mouse from line 74 shows declined performance on the treadmill over a period of 6 days. The videos were recorded on P30 (top), P35 (middle), and P41 (bottom). Note that the treadmill is held at different speeds. Click here for file

Additional file 6 **Analysis of p23 and synaptic marker expression**. Double immunofluorescence analysis of calbindin and VGLUT1 or VGLUT-2 labeling in cerebellum of non-transgenic animals. The following areas are indicated: *Pcl*, Purkinje cell layer; *gcl*, granule cell layer, *ml*, molecular layer. Click here for file
