# Supplementary figures and images for: Transgenic neuronal overexpression reveals that stringently regulated p23 expression is critical for coordinated movement in mice
Source: Mol Neurodegener. 2011 Dec 28;6:87. doi: 10.1186/1750-1326-6-87 (PMC3259059; doi:10.1186/1750-1326-6-87)

Additional File 6

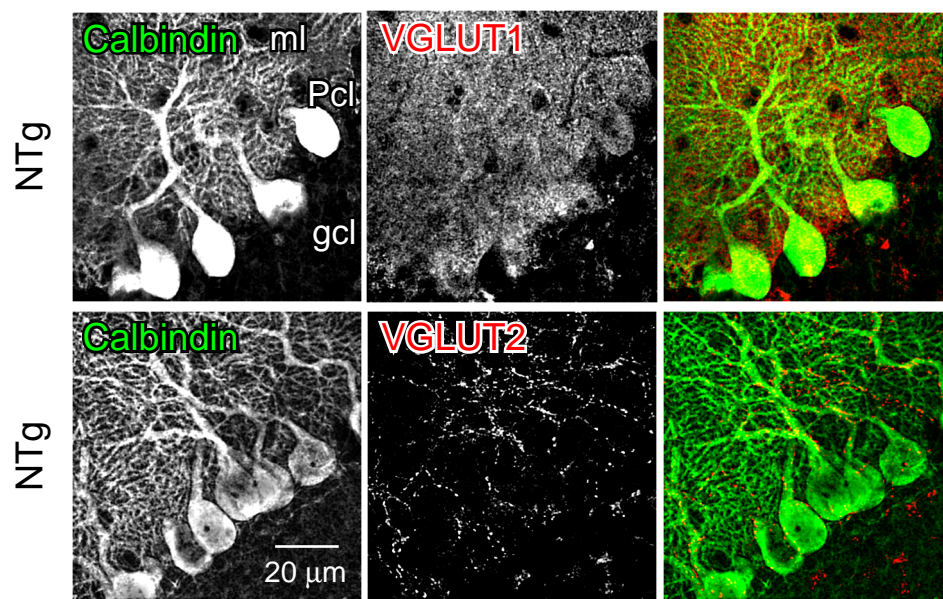

Supplement: Additional file 6 — Analysis of p23 and synaptic marker expression. Double immunofluorescence analysis of calbindin and VGLUT1 or VGLUT-2 labeling in cerebellum of non-transgenic animals. The following areas are indicated: Pcl, Purkinje cell layer; gcl, granule cell layer, ml, molecular layer. [file 1750-1326-6-87-S6.PDF]
